# A Retrospective Observational Study to Assess the Effect of the COVID-19 Pandemic on Spontaneous and Voluntary Abortivity in the Apulia Region of Italy

**DOI:** 10.3390/life13010120

**Published:** 2022-12-31

**Authors:** Ilaria Dargenio, Nicola Bartolomeo, Massimo Giotta, Maria Elvira Metta, Paolo Trerotoli

**Affiliations:** Interdisciplinary Department of Medicine, University of Bari Aldo Moro, 70121 Bari, Italy

**Keywords:** COVID-19, abortion rate, voluntary termination of pregnancy, restrictive public health measures, Poisson counting model

## Abstract

The spread of COVID-19 in Italy required urgent restrictive measures that led to delays in access to care and to hospital overloads and impacts on the quality of services provided by the national health service. It is likely that the area related to maternal and child health was also affected. The objective of the study was to evaluate the intensity of a possible variation in spontaneous abortion (SA) and voluntary termination of pregnancy (VTP) rates in relation to the different restrictive public health measures adopted during the pandemic period of 2020. The analysis concerned the data collected on the SAs and VTPs from public and private structures in Apulia that related to the years 2019 and 2020. The SRR (standardized rate ratio) between the standardized rates by age group in 2019 and those in 2020 were calculated using a multivariable Poisson model, and it was applied to evaluate the effect of public health restrictions on the number of SAs and VTPs, considering other possible confounding factors. The SSR was significantly lower in the first months of the pandemic compared to the same period of the previous year, both for SAs and for VTPs. The major decrease in SAs and VTPs occurred during the total lockdown phase. The results, therefore, highlight how the measures to reduce infection risk could also have modified the demand for assistance related to pregnancy interruption.

## 1. Introduction

On 30 January 2020, the WHO declared that the international outbreak of new Coronavirus was a public health emergency of international concern (PHIEC) [1]. The SARS-CoV-2 infection, identified in the city of Wuhan, spread to the rest of China and around the world, assuming pandemic characteristics. The spread of COVID-19 in Italy required urgent restrictive measures, and it was necessary to control the diffusion of infections, and to manage hospital overload in the Italian national health system (INHS). The public health measures may have led to delays in access to care and to hospital overload, as well as to a social impact on patients’ mental and psychological well-being, resulting in a domino effect that caused a decrease in the quality of most of services provided by the INHS. Among these services, it is likely that the areas related to maternal and child health were also affected, as several studies have shown [2,3].

Advisory services related to this area, as recommended by the American College of Obstetricians and Gynecologists and the American Academy of Pediatrics, should have been provided without discontinuance as part of social health planning services [4,5]. Nevertheless, in Texas, Louisiana, and in most of the United States of America, in order to slow the spread of SARS-CoV-2 infection, services for both surgical abortions and pharmacological abortions were stopped; meanwhile, in other northern states of America, by adopting other public health strategies, access to surgical abortion was reduced exclusively, allowing for pharmacological abortions only [6]. In the European Union, on the other hand, activities related to voluntary termination of pregnancy (VTP) were discontinued in six countries (Andorra, Liechtenstein, Malta, Monaco, San Marino, and Poland), and suspended in Hungary. Access to surgical VTPs was severely restricted in 12 European countries and denied altogether to women with symptoms of COVID-19 infection in 11 countries (the Netherlands, Belgium, Germany, Iceland, Latvia, Luxembourg, Montenegro, Slovenia, England, Wales, and Scotland) [7]. Seven countries organized the service for assisted pharmacological VTP through a telemedicine program. In Denmark and Sweden, this possibility already existed before the pandemic, and five other countries introduced it (England, Wales, Scotland, France, and Ireland). Eight countries provided home medical VTP, with mifepristone (RU486) and misoprostol, beyond 9 weeks (up to 11 weeks + 6 days) [7]. In Italy, the new restrictive public health policies during the lockdown highlighted the vulnerability of health services, including access to VTP, a right guaranteed by Law 194/78. In 2019, 73,207 VTPs were carried out in Italy, with an abortion rate of 5.8 VTPs per 1000 women between 15 and 49 years of age. For 2020, 67,638 voluntary terminations of pregnancy were reported, with an abortion rate of 5.5 VTPs per 1000 women [8].

These results may have occurred because it was decided to postpone planned interventions, and only at the end of March 2020 did the Ministry of Health clarify that the services related to VTPs would not be postponed; however, hospitals resumed this slowly [9]. 

The INHS did, therefore, not ensure a clear path for essential and urgent care. Further difficulties arose due to general lockdown measures, such as unavailability of transport services and prohibition to move from the home [10].

Another side of the assistance to women that tended to interrupt pregnancy was the availability of family counseling centers (FC). These have a key role in the protection of maternal and child health, as well as to the development of conscious choices related to procreation and parenting; since their establishment with Law 405/1975 [11], Law no. 34/96 provides for the presence of an FC for at least every 20,000 inhabitants, considering that a free and open access service cannot be provided to larger target populations [12].

The FCs also provide assistance post-abortion, but as shown in The Annual Report to Parliament on VTP in Italy [8], a decrease in post-abortion control visits carried out during the pandemic period was registered, even if post-abortion controls represent a fundamental family planning tool to limit and avoid the use of further abortions [13]. It was assumed, however, that the pandemic altered the territorial equilibrium through the increase in the catchment areas of FCs, due to temporary closures of some of them. 

The pandemic has, therefore, shown the need to strengthen the local health system to reduce dependency on hospital services [14].

In addition, various other structural criticalities were highlighted due to the absence of specialists during the lockdown. Several studies verified an increase in unwanted pregnancies, probably related to forced critical cohabitation conditions of domestic life [2,3]. Furthermore, it could be argued that the economic and financial difficulties caused by the pandemic contributed to this increase in interrupted pregnancies [15]. 

Particularly strong drops in birth rate occurred in southern Europe in the year 2021 compared to 2016, in Italy (−9.1%), Spain (−8.4%) and Portugal (−6.6%) [16]. This could be due to a decline in nuptiality resulting from the restrictive measures, as a positive correlation existed between the two events. Furthermore, employment shortages, combined with economic hardships and the condition of psychological and financial uncertainty, seem to have determined decisions to postpone possible pregnancies [17].

This combination of social conditions that could have affected birth rate may have had an indirect effect on spontaneous abortions (SAs), as with fewer pregnancies, there are fewer miscarriages. This happens in 7.55 per 1000 pregnancies per year in Italy [18], and in 2019 there were 48,932 spontaneous abortions reported in Italy, which decreased to 41,493 in 2020 [19].

In this retrospective study, the abortion rates related to voluntary terminations of pregnancy (VTPs) and spontaneous abortions (SAs) of 2020 were compared with those of 2019. The main objective was to assess the extent of a possible variation in the rates and its relationship with the different phases of the pandemic that were characterized by different levels of restrictions to public health services adopted during the pandemic period of 2020.

## 2. Materials and Methods

A retrospective observational study was performed on SAs and VTPs that occurred in Apulia to evaluate abortion rates during pandemic. Data were collected according to the regulations established each year by the National Surveillance System, managed by the National Institute of Statistics (ISTAT), and the data were related to VTPs and SAs that occurred in public and private health institutes. Specific data of this study were taken from the Health Information System of the Apulia region.

The data were treated according to the principles of correctness, lawfulness, transparency, and pursuant to Article 24 of Regulation (EU) 2016/679.

The analysis concerned data of public and private structures in Apulia that related to the period from January 1st 2019 to December 31st 2020. Patients aged over 50 and women who did not provide their birthdates were excluded from the study. The patients were not allowed to put any type of identification or sensitive data into the form, even if health personnel were obliged to report the event [20,21]. The crude abortion rates for the year 2019 and the year 2020 were calculated as the number of events (SAs or VTPs) divided by the respective average populations of women between 9 and 49 years; the numbers were determined using the official data made available by the ISTAT [22]. Rates per 10,000 women were calculated monthly, and were subsequently standardized by age group (<15 years, 15–19 years, 20–24 years, 25–29 years, 30–34 years, 35–39 years, 40–44 years, and 45–49 years [8]) with the direct method, using the Apulian population at 1 January 2020 as the standard population. The SRR (standardized rate ratio) was calculated by dividing the standardized abortion rates of 2020 by those of 2019. A 95% confidence interval was calculated, under the assumption of a normal sample distribution of the log (SRR).

A multivariable Poisson regression model was used to investigate the possible effects of the pandemic on the variation in the numbers of SAs and VTPs. To this end, the analysis period, from 1 January 2019 to 31 December 2020, was divided into four phases, with different levels of restrictions; it has already been shown that these phases had an effect on health outcomes, such as in-hospital mortality [23]:

1. Phase 1, total lockdown, from 1 March 2020 to 30 April 2020, with a high level of restrictions, including prohibition to leave home, except for reasons of necessity, suspension of educational services for children and schools of all orders and degrees, and suspension or limitation of the performance of work activities, favoring the performance of smart working [24].
2. Phase 2, medium-level lockdown, from 1 May 2020 to 15 June 2020, and from 1 October 2020 to 31 December 2020, with a moderate level of restrictions, including prohibition to move from one’s own municipality of residence, except for reasons of necessity, suspension of congress, sports competitions, and commercial activities, and closure of restaurants, gyms, sports centers, museums, and places of culture [25,26].
3. Phase 3, from 16 June 2020 to 30 September 2020, with a low level of restrictions, during which access to numerous activities could take place with organizational measures to avoid gatherings of people, taking into account the size and characteristics of the places [27].
4. Phase 4, from 1 January 2019 to 29 February 2020, without any restrictions or limitations.

The count of events in the different pandemic phases was estimated with a model for SA and a model for VTP, including as predictors the chronological age, the gestational age of the patient, and the number of previous SAs or previous VTPs. The age was classified into seven classes by combining, with respect to standardization, the women under 15 and women between 15–19 years into a single class. All other variables were classified using the same classes as those in the annual report [8]. The gestational age was expressed through the classes of completed weeks of amenorrhea, as follows: ≤8 weeks, 9–10 weeks, 11–12 weeks, and ≥13 weeks. Previous SAs and VTPs were divided into five classes, as follows: no previous abortions, one previous abortion, two previous abortions, three previous abortions and four or more previous abortions.

To avoid an excessive loss of information, women who did not provide information on gestational age were also included in the Poisson model; using the statistical technique of missing imputation, a predictive analysis allowed estimation of the missing gestational age through information obtained from other variables. Successively, we first applied a generalized linear regression model to the complete data, in order to evaluate the variables that were predictive of gestational age; then, the estimated values were used for the missing imputation procedure. The age, the number of previous SAs, the type of intervention performed after the SA, the number of hospital days, the possible presence of complications, and the occupational status, marital status, and citizenship resulted as significant predictors to estimate gestational age. 

The variables used for the missing imputation of the gestational age in the VTP data were as follows: age, type of intervention practiced for the VTP, type of analgesic therapy, the number of days of hospitalization, the occupational status, the marital status, and educational qualifications.

In the Poisson model, as recommended by Cameron and Trivedi [28], we used robust standard errors for the parameter estimates in order to check for the violation of the distribution assumption that the variance was equal to the mean.

The results of the Poisson model are shown as the incidence rate ratio (IRR) between the different levels of the covariates and the respective 95% confidence intervals. The IRRs were obtained by calculating the exponential of the estimated parameters. Multiple pairwise comparisons were adjusted through Tukey’s method.

Results with *p* < 0.05 were considered statistically significant. The statistical analyses were developed with version 4.1.2 of software R [29], with the packages “car” [30], “dplyr”, [31] and “emmeans” [32].

## 3. Results

There were 11,474 patients who underwent a VTP, of which 6162 (6.4 per 1000) were from 2019 and 5312 (5.7 per 1000) were from 2020; only 2 women were removed from the analysis because of non-reliable ages (>50); 8 more women were removed (1 in 2019 and 7 in 2020) because they did not give consent to use personal data, so age could not be determined. The mean (sd) age of women that underwent a VTP was 30.6 (7.4).

Spontaneous abortion occurred in 7765 patients, of which 4146 (4.3 per 1000) were from 2019 and 3619 (3.9 per 1000) were from 2020. Women aged over 50 were removed from the analysis, of which there were 7 in 2019 and 6 in 2020. The mean (sd) age of women that had an SA was 34.3 (6.3).

The main characteristics of the patients included in the study, grouped by the year in which the event occurred, are summarized in Table 1, both for SAs and VTPs.

### 3.1. Voluntary Termination of Pregnancy (VTP)

In 2019, the absolute lowest number of VTPs was 397 in August, with a standardized abortion rate of 4.14 per 10,000 women; the highest was 634 in January, with a standardized abortion rate of 6.61 per 10,000 women. In 2020, the lowest absolute number of VTPs was 350 in November, with a standardized abortion rate of 3.73 per 10,000 women, while the highest was 549 in January, with a standardized abortion rate of 5.84 per 10,000 women.

**Figure 1:**
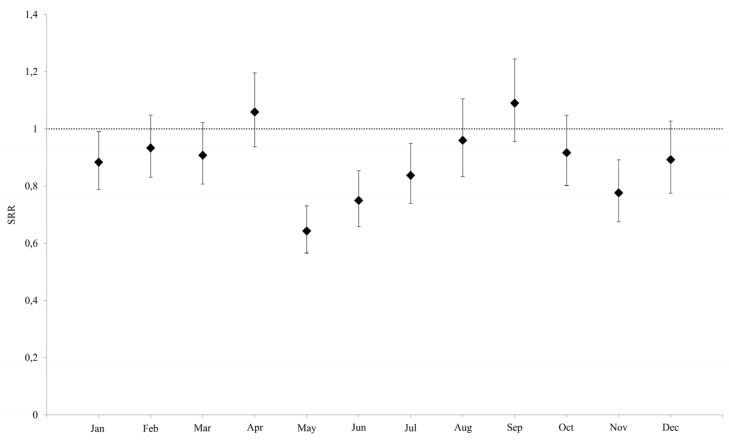
Standard rate ratio (IC 95%) of VTPs in 2020 in Apulia, as compared to the rate of Apulia in 2019.

The decrease in VTPs from 2019 to 2020 was statistically significant in the months of January, May, June, July, and November (Figure 1).

The lowest SRR was that of May (SRR: 0.643, 95% CI 0.566–0.731), in which the decrease in VTPs was particularly significant in patients in the age class of 15–19 years (SRR: 0.318, 95% CI 0.182–0.558). Following this were the months of June (SRR: 0.750, CI95% 0.659–0.854) and November (SRR: 0.776, CI95% 0.676–0.892) (Table 2).

The effects of the phases related to pandemic restrictions on VTPs were evaluated through the Poisson multivariable regression model, including as covariates the age class, the class of weeks of amenorrhea performed, and the class of previous VTPs.

**Figure 2:**
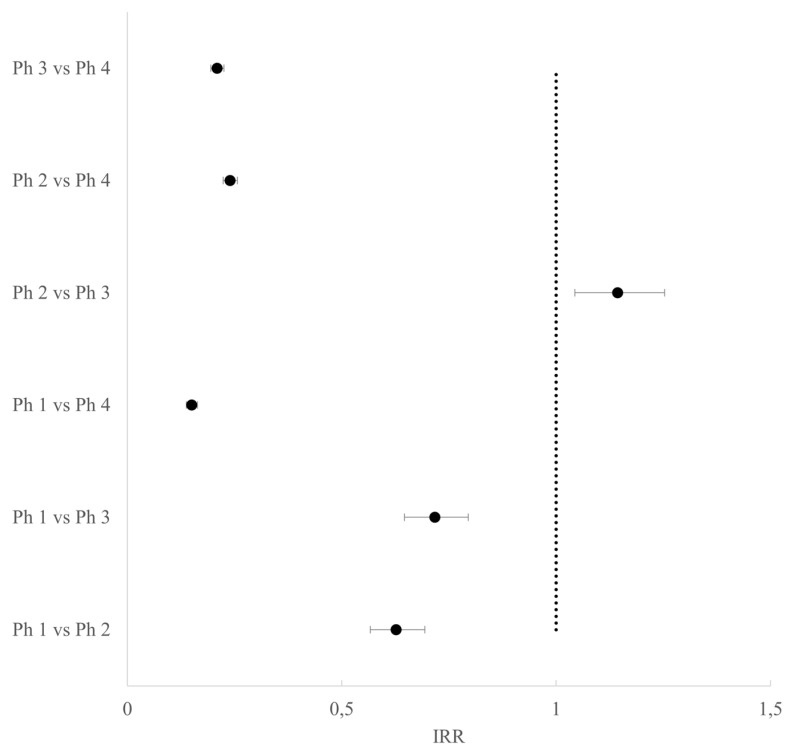
Forest plot of the incidence rate ratios and their adjusted 95% CI for VTPs between phases.

According to the test scores for type III contrasts, all covariates were statistically significant (for all, the *p*-value was < 0.0001). There was a decrease in events in all phases characterized by restrictions compared to Phase 4 (pre-lockdown), particularly during Phase 1 compared to Phase 4 (IRR 0.15, 95% CI 0.14–0.16). In Phase 1, the number of VTPs was also significantly lower than those in Phases 2 and 3, while in Phase 2 it was slightly higher than that in Phase 3 with fewer restrictions (Figure 2 and Table S1).

Women of all age groups, except those in the 45–49 years class, showed a statistically significant increase in VTPs compared to younger women (aged ≤ 19 years), particularly in the 30–34 years age group (IRR 2.53, 95% CI 2.26–2.84) and in the age group 35–39 (IRR 2.53, 95% CI, 2.25–2.83).

Women at any time of gestational age showed a statistically significant decrease in VTP compared to women with shorter gestational age (≤8 weeks of amenorrhea), particularly those with amenorrhea weeks between 11 and 12 (IRR 0.11, 95% CI 0.1–0.12), and those with amenorrhea weeks ≥13 (IRR 0.11, 95% CI 0.1–0.13).

Furthermore, all women who had at least one previous VTP showed a statistically significant decrease compared to women who never had a VTP previously, particularly those who had three previous voluntary abortions (IRR 0.04, 95% CI 0.03–0.05), and those who had four or more previous voluntary abortions (IRR 0.03, 95% CI 0.02–0.03) (Table S1).

### 3.2. Spontaneous Abortions (SA)

In 2019, the lowest absolute number of SAs was recorded in September (248), with a standardized abortion rate of 2.58 SAs per 10,000 women aged 9–49, while the highest number (410) occurred in March, with a standardized abortion rate of 4.27 per 10,000 women. In 2020, the lowest absolute number of SAs was 251 in November, with a standardized abortion rate of 2.68 per 10,000 women, while the highest (396) was recorded in January, with a standardized abortion rate of 4.22 per 10,000 women.

**Figure 3:**
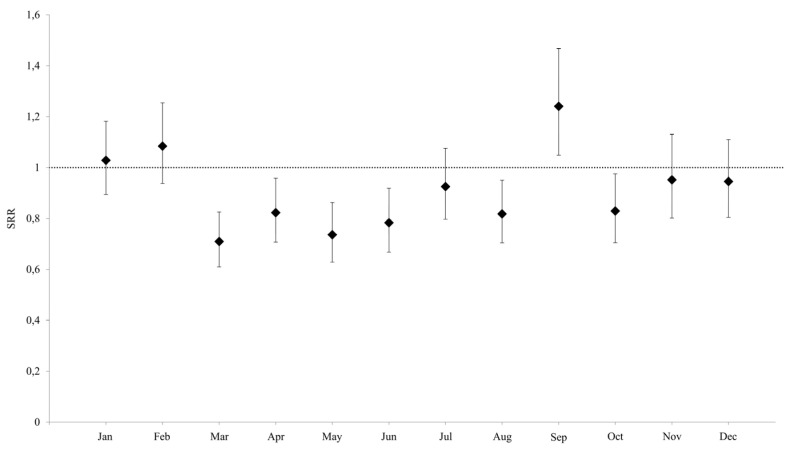
Standard rate ratio (IC 95%) of spontaneous abortions in 2020 in Apulia, as compared to the rate of Apulia in 2019.

Comparing the monthly distribution of SRRs between 2019 and 2020, there was a statistically significant reduction in the number of events in the months of March, April, May, June, August, and October. The lowest SRR was that of March, with an SRR of 0.710 [95% CI 0.61–0.826], followed by the months of May (SRR: 0.736, 95% CI 0.628–0.863) and June (SRR 0.783, 95% CI 0.668–0.919). In contrast, for the SRR of September, the number of SAs detected in 2020 was greater than the previous year (SRR 1.240, CI95% 1.048–1.468) (Figure 3).

As of March 2020, the decrease in SA is significant in patients between 30 and 34 years (SRR 0.650, 95% CI 0.477–0.885) and in those between 40 and 44 years (SRR 0.601, 95% CI 0.432–0.835). Furthermore, in May, the SRR was significantly lower than 1 in the same age groups, and in general the 40–44 years class is the one where the decrease in SA in the months of 2020 was more frequent. The only SRR significantly higher than 1 was recorded in September in the 35–39-year-old class. (Table 3).

Through the Poisson multivariable regression model, the effect of the phases related to pandemic restrictions on the phenomenon of abortion was evaluated, inserting as covariates the age class, the class of weeks of amenorrhea performed, and the class of previous SAs.

**Figure 4:**
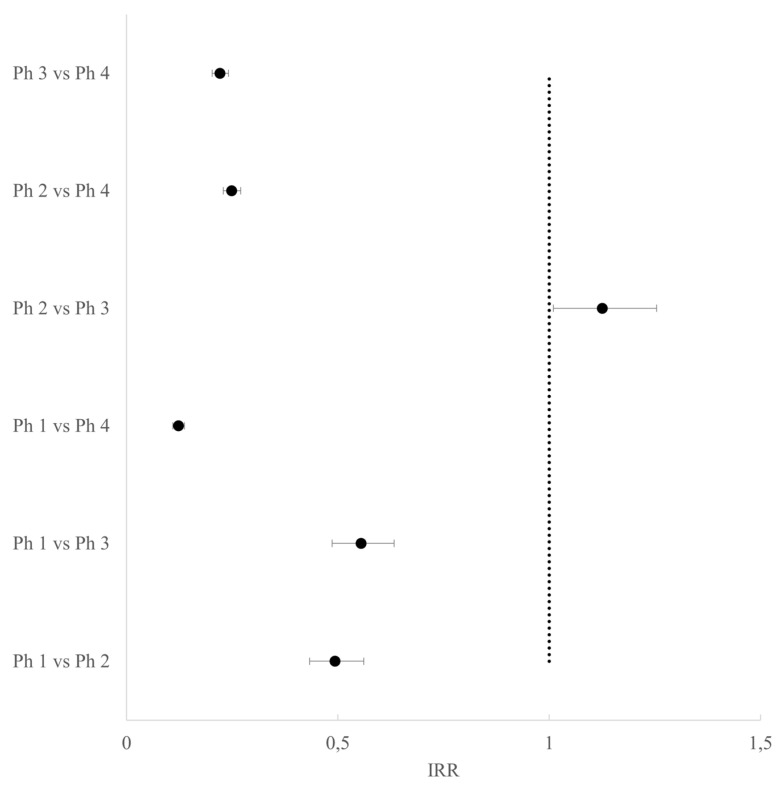
Forest plot of the incidence rate ratios and their adjusted 95% CI for SAs between phases.

According to the score test for type III contrasts, all covariates were statistically significant (for all, the *p*-value was < 0.0001). There was a decrease in events in all phases characterized by restrictions compared to Phase 4 (pre-lockdown), in particular during Phase 1 as compared to Phase 4 (IRR 0.12, CI95% 0.11–0.14). In Phase 1, the number of SAs was also significantly lower than in Phases 2 and 3, while in Phase 2 it was slightly higher than in Phase 3 with fewer restrictions (Figure 4 and Table S2).

Women of all age groups showed a statistically significant increase in SAs compared to younger women (aged ≤19 years), particularly in the age group 30–34 years (IRR 13.17, 95% CI 10.09–17.2), and in the age group 35–39 (IRR 14.84, 95% CI, 11.38–19.37). Women at any time of gestational age showed a statistically significant decrease in SAs compared to women with gestational age in the lowest class (≤8 weeks of amenorrhea), particularly those with 11 to 12 weeks of amenorrhea (IRR 0.34, 95% CI 0.31–0.37), and those with weeks of amenorrhea ≥13 (IRR 0.24, 95% CI 0.22–0.27). Women who had had at least one previous SA showed a statistically significant decrease in SAs compared to women who had never had a previous SA, particularly those who had three previous miscarriages (IRR 0.03, 95% CI 0.02–0.04), and those who had four or more previous spontaneous abortions (IRR 0.02, 95% CI 0.02–0.03) (Table S2).

## 4. Discussion

In this study we analyzed the trends in spontaneous (SA) and voluntary (VTP) abortions in the period 2019–2020 in order to evaluate the effects of the restrictive measures adopted during the COVID-19 pandemic. The study was conducted in Apulia, an Italian region with around 800,000 women of childbearing age. Regarding SAs, the decrease in events was evident from the beginning of the pandemic and corresponded to the total lockdown, and it is precisely in this phase that the greatest decrease was recorded, both compared to the pre-pandemic period and to other phases with less stringent levels of restrictions. The decrease in events was more significant during Phase 1, “total lockdown”, for VTP events as well. A decline in the number of events managed by maternal and child departments occurred, although a good number of health facilities and abortion centers remained operational and guaranteed their activities during the pandemic.

The pandemic may have had a double effect on indirect reduction in SAs, namely fewer pregnancies because of reduced contact from fear of infection, and a consequent decrease in SAs, as well as increased use of contraceptive methods to avoid pregnancies and the risks of going to the hospital. This latter reason may have increased a more consistent promotion of contraception [33]. Furthermore, Hedermann et al. showed that there was a reduction in the number of premature births during the pandemic lockdown [34], and this may be evidence of how the pandemic affected pregnancies and outcomes of pregnancies.

An Italian study, conducted in Piedmont, did not find an increased risk of SAs during the pandemic in pregnant women who had contracted the SARS-CoV-2 infection during the first trimester of pregnancy [35]; this is the period with a higher risk of miscarriage. According to Hedermann et al. [34], the lockdown due to COVID-19 drastically changed our lives by reducing physical interactions, through physical distancing and home confinement, increasing our attention to hygiene, changing our work environment, and by reducing atmospheric pollution levels. It is likely that this unusual situation reduced the influence of various risk factors that could have had a negative impact on the maternal–infant area. A high number of spontaneous abortions appears to be caused by infectious origins that alter the general inflammatory state of pregnant women. 

The COVID-19 restriction measures (lockdown, use of face masks) had an effect in the significant decrease observed in the incidence of influenza and other viral and bacterial infections [36], which could partially explain the reduction in the number of SAs, especially for those caused by infections; moreover, this reduction appeared particularly during the most restricted phase.

The decrease in VTPs may have been caused by conflicting messages from social media and other communication networks about accessibility to services related to the maternal–infant area, and by the fear of contracting the virus in health facilities [37]. 

Italy was the first of the European Union countries to have been intensely affected by the health emergency from COVID-19, which highlighted shortcomings in an Italian health system that was unprepared to face a massive demand for services. The response was to delay or to close many services, and this, together with the fear of infection, may have led a part of the population to avoid calling for VTPs. Our results showed a decrease in VTPs, precisely during the phases that were characterized by restrictions on interregional mobility. Many issues beyond the pandemic affected the access to VTPs. There were elevated numbers of health workers that applied conscientious objection (CO), especially in southern regions, such as Apulia, in which CO is chosen by 80% of physicians. Actually, in 2019, the European Committee of Social Rights (CEDS) urged the Italian government to guarantee a more homogeneous distribution of non-objecting personnel, particularly in deficient southern regions [38]. It is possible to hypothesize that the constant decrease in health personnel, linked to the numerous infections among health workers, emphasized the problematic access to VTPs linked to the high numbers of gynecologists, anesthetists, and health professionals who were conscientious objectors [39]. Thus, women had to face limited access to FCs for VTPs, due to the unavailability of personnel, and during the pandemic, due to closures of health care facilities related to VTPs.

The pandemic has led to an economic crisis, but previous studies have not shown a relationship between socio-economic deprivation and TVP rates. A study on abortion rates observed before and during the economic crisis of 2008 showed that the crisis did not change the rate trend, nor the relationship between rates and deprivation. The crisis had only increased and made more evident the disparities in access to health services [40]. The difficulty in accessing health services is a very serious problem even in scenarios of internal conflicts or war. In these conditions, violence causes unwanted pregnancies, and the weakness of the national organization causes difficulties in accessing health services, including those for maternal health, abortion, and contraception [41].

The restrictions related to the pandemic also had a negative influence on social dynamics compared to the pre-pandemic period; in particular, the southern regions, such as Apulia, were characterized by a strong migratory flow for study or work towards the northern regions. Those who lived at a distance, also thanks to distance learning or smart working, returned to live with their families, intensifying the co-presence of parents and children in the same home for longer times than usual. This cohabitation in many cases may have favored and facilitated intra-family and intra-marital communication, which could have been a determining factor in a woman’s reproductive choices. According to the study by Ituarte et al. of 2021, the parental figure, especially the maternal figure, played an essential role in decisions to voluntarily terminate pregnancies [42]. Our hypothesis is that the alteration of family/marital and social/communication balances influenced the choices made by women.

To respond to the pandemic, the Italian government and health personnel addressed the question of how to continue providing essential health services and concluded that telemedicine could be the right application. The COVID-19 emergency led some health systems to re-evaluate the concept of telemedicine, by recognizing the importance of medical consultations regarding abortion and the subsequent completion of pharmacological abortion, and by sending abortion pills (mifepristone) by mail [43]. This experience, already applied in the United Kingdom, could perhaps lead other states to understand how direct control over every health intervention can be replaced, at least in cases of emergency. Furthermore, self-management by the user would not mean an absence of medical assistance, but a planned and more practical care of patients. In Australia, the government expanded telemedicine services during the lockdown. Telemedicine consultations for early medical abortion increased by 25% since the start of the pandemic, indicating that telemedicine services can improve the quality of services offered. Furthermore, the use of telemedicine services in Australia has eliminated the fear of contagion and has been shown to have alleviated pressure on troubled health systems [44]. In Ethiopia, the government approved a pilot program to allow voluntary abortions directly at home during the lockdown period. In Italy, on the other hand, this alternative route did not appear to have been sufficiently followed, given the decrease in voluntary abortions [45], in accordance with our data. Although limited to the pandemic period, these declines can reduce the impact on these services which are offered by the INHS and could dampen the future effectiveness of family planning and health education programs, especially in the maternal–child area.

In light of the different degrees of incidence of COVID-19 and the different organization strategies adopted by health services, further studies concerning other geographical areas, besides the Apulian region, could allow for comparisons between regions and their strategies to face VTP demands from their populations.

Furthermore, an analysis of social and economic determinants, such as deprivation, could constitute a subsequent study to evaluate how the restrictive measures led to a reduction in conceptions as a result of reductions in social contact, with an indirect effect on abortion, especially in weaker strata of the population, and in young women [46].

## 5. Conclusions

Our study showed a possible association between voluntary/spontaneous abortion and restrictive public health measures. The decrease in VTP events may be related to the lower availability of health services. The decrease in SAs may be explained by indirect causes, such as reductions in the birth rate mainly linked to financial as well as logistical difficulties, by changes in the behaviors of women and men, and by public health preventive measures. Further studies could confirm the results reported here and suggest approaches to organizing health services during emergencies, such as pandemics, in order to ensure care for women on the sensitive path of pregnancy.

## Figures and Tables

**Table 1:** Distribution by age group, amenorrhea week class, previous SA or previous VTP class, and pandemic phase; SA and VTP events, years 2019 and 2020, Apulia region.

Parameter	SA						VTP					
	2019		2020		Total		2019		2020		Total	
	N	%	N	%	N	%	N	%	N	%	N	%
Age class												
≤19	70	1.7	60	1.7	130	1.7	523	8.5	381	7.2	904	7.9
20–24	286	6.9	220	6.1	506	6.5	1018	16.5	875	16.5	1893	16.5
25–29	569	13.7	520	14.4	1089	14.0	1283	20.8	1016	19.1	2299	20.0
30–34	1078	26.0	947	26.2	2025	26.1	1296	21.0	1161	21.9	2457	21.4
35–39	1153	27.8	1133	31.3	2286	29.4	1262	20.5	1193	22.5	2455	21.4
40–44	869	21.0	650	18.0	1519	19.6	709	11.5	610	11.5	1319	11.5
45–49	121	2.9	89	2.5	210	2.7	71	1.2	76	1.4	147	1.3
Amenorrhea (weeks)												
≤8	1730	41.7	1435	39.7	3165	40.8	3837	62.3	3231	60.8	7068	61.6
9–10	1518	36.6	1313	36.3	2831	36.5	1628	26.4	1405	26.4	3033	26.4
11–12	531	12.8	506	14.0	1037	13.4	327	5.3	356	6.7	683	6.0
≥13	367	8.9	365	10.1	732	9.4	370	6.0	320	6.0	690	6.0
Previous abortions (number)												
0	2961	71.4	2664	73.6	5625	72.4	4381	71.1	3783	71.2	8164	71.2
1	817	19.7	651	18.0	1468	18.9	1149	18.6	1019	19.2	2168	18.9
2	251	6.1	195	5.4	446	5.7	403	6.5	336	6.3	739	6.4
3	76	1.8	71	2.0	147	1.9	147	2.4	110	2.1	257	2.2
≥4	41	1.0	38	1.1	79	1.0	82	1.3	64	1.2	146	1.3
Phase												
1	0	0.0	583	16.1	583	7.5	0	0.0	1048	19.7	1048	9.1
2	0	0.0	1200	33.2	1200	15.5	0	0.0	1689	31.8	1689	14.7
3	0	0.0	1066	29.5	1066	13.7	0	0.0	1484	27.9	1484	12.9
4	4146	100.0	770	21.3	4916	63.3	6162	100.0	1091	20.5	7253	63.2

**Table 2:** Standard rate ratio (95% CI) of VTPs between 2020 and 2019, by age group and month, Apulia region.

Month	Age Class (Years)							
	<15	15–19	20–24	25–29	30–34	35–39	40–44	45–49
Jan	-	0.68 [0.45–1.02]	0.92 [0.69–1.21]	**0.74** **[0.57–0.97]**	1.11 [0.88–1.41]	0.95 [0.73–1.22]	0.72 [0.52–1.01]	1.51 [0.42–5.34]
Feb	-	1.06 [0.7–1.61]	1.01 [0.75–1.35]	0.9 [0.69–1.17]	0.95 [0.74–1.22]	0.84 [0.66–1.08]	1 [0.71–1.39]	0.86 [0.29–2.56]
Mar	-	1.1 [0.69–1.76]	0.93 [0.7–1.23]	0.78 [0.59–1.02]	0.93 [0.72–1.2]	0.93 [0.72–1.2]	0.99 [0.71–1.36]	0.5 [0.15–1.67]
Apr	-	1.1 [0.69–1.76]	0.83 [0.6–1.14]	1.19 [0.91–1.55]	1.21 [0.93–1.56]	1.02 [0.79–1.32]	0.94 [0.65–1.35]	1.23 [0.51–2.96]
May	2.03 [0.37–11.1]	**0.32** **[0.18–0.56]**	**0.59** **[0.42–0.82]**	**0.52** **[0.39–0.69]**	**0.69** **[0.53–0.91]**	**0.74** **[0.57–0.97]**	0.95 [0.66–1.38]	0.5 [0.09–2.74]
Jun	-	**0.46** **[0.28–0.75]**	**0.67** **[0.48–0.92]**	**0.71** **[0.53–0.95]**	0.85 [0.65–1.13]	0.84 [0.63–1.12]	0.78 [0.54–1.14]	1.15 [0.42–3.16]
Jul	-	**0.56** **[0.33–0.97]**	1.11 [0.83–1.5]	0.83 [0.63–1.1]	**0.67** **[0.51–0.89]**	0.89 [0.68–1.16]	0.77 [0.53–1.1]	2.61 [0.93–7.32]
Aug	2.03 [0.18–22.4]	0.76 [0.46–1.25]	1.04 [0.72–1.5]	**0.55** **[0.4–0.77]**	1.11 [0.81–1.51]	1.25 [0.93–1.67]	1.16 [0.77–1.74]	0.5 [0.09–2.74]
Sep	-	0.88 [0.55–1.41]	0.96 [0.7–1.31]	1.05 [0.78–1.41]	0.95 [0.7–1.27]	1.45 [1.1–1.92]	1.16 [0.77–1.77]	1.15 [0.42–3.16]
Oct	0.51 [0.05–5.61]	**0.61** **[0.4–0.94]**	**0.68** **[0.49–0.94]**	0.98 [0.73–1.31]	0.9 [0.67–1.22]	1.28 [0.94–1.75]	1.04 [0.7–1.54]	1.67 [0.4–7]
Nov	-	0.87 [0.53–1.42]	0.82 [0.59–1.13]	**0.72** **[0.53–0.98]**	**0.67** **[0.49–0.91]**	1.01 [0.74–1.36]	**0.6** **[0.38–0.94]**	0.67 [0.24–1.88]
Dec	0.51 [0.09–2.78]	0.78 [0.49–1.26]	0.93 [0.66–1.31]	0.96 [0.7–1.31]	0.95 [0.71–1.27]	0.84 [0.61–1.15]	0.79 [0.5–1.26]	1.25 [0.34–4.67]

Statistically significant SSRs are in bold.

**Table 3:** Standard rate ratio (95% CI) of SAs between 2020 and 2019, by age group and month, Apulia region.

Month	Age Class (Years)							
	<15	15–19	20–24	25–29	30–34	35–39	40–44	45–49
Jan	-	0.3 [0.08–1.11]	1.09 [0.7–1.69]	1.23 [0.87–1.75]	1.16 [0.87–1.55]	0.94 [0.73–1.22]	0.98 [0.7–1.38]	0.55 [0.2–1.48]
Feb	-	0.71 [0.27–1.87]	0.87 [0.48–1.59]	1.01 [0.7–1.44]	1.24 [0.94–1.64]	1.55 [1.16–2.07]	**0.68** **[0.48–0.96]**	0.88 [0.32–2.42]
Mar	-	2.03 [0.69–5.94]	0.59 [0.32–1.07]	0.75 [0.51–1.11]	**0.65** **[0.48–0.88]**	0.81 [0.61–1.08]	**0.6** **[0.43–0.83]**	0.78 [0.29–2.1]
Apr	-	1.52 [0.43–5.4]	0.63 [0.35–1.16]	0.74 [0.49–1.13]	0.77 [0.58–1.03]	0.91 [0.69–1.21]	0.81 [0.57–1.15]	1.23 [0.51–2.96]
May	-	0.29 [0.06–1.4]	0.57 [0.29–1.12]	1.05 [0.69–1.58]	**0.64** **[0.47–0.86]**	0.99 [0.73–1.36]	**0.61** **[0.43–0.86]**	0.4 [0.16–1.03]
Jun	-	0.76 [0.17–3.4]	0.75 [0.41–1.39]	1 [0.67–1.5]	**0.7** **[0.51–0.95]**	0.84 [0.63–1.13]	0.74 [0.5–1.09]	0.42 [0.15–1.19]
Jul	-	2.03 [0.61–6.74]	0.91 [0.49–1.69]	0.98 [0.66–1.46]	1 [0.73–1.37]	1.05 [0.8–1.38]	**0.72** **[0.52–0.99]**	0.43 [0.17–1.12]
Aug	-	-	0.67 [0.36–1.26]	1.08 [0.69–1.68]	0.86 [0.63–1.16]	0.95 [0.74–1.22]	**0.55** **[0.39–0.78]**	1.38 [0.56–3.43]
Sep	-	0.15 [0.02–1.18]	0.92 [0.41–2.09]	1.02 [0.62–1.68]	1 [0.73–1.37]	**1.72** **[1.25–2.37]**	1.35 [0.93–1.94]	1.51 [0.62–3.68]
Oct	-	1.62 [0.53–4.96]	0.78 [0.41–1.46]	**0.58** **[0.37–0.89]**	0.97 [0.71–1.34]	0.78 [0.58–1.06]	0.89 [0.62–1.28]	0.88 [0.32–2.42]
Nov	-	2.03 [0.61–6.74]	0.75 [0.39–1.47]	1.02 [0.63–1.66]	1.04 [0.74–1.47]	0.98 [0.73–1.32]	0.8 [0.52–1.23]	0.45 [0.14–1.45]
Dec	-	2.03 [0.37–11.1]	0.7 [0.37–1.32]	0.87 [0.56–1.37]	0.87 [0.65–1.18]	1.04 [0.77–1.4]	1.08 [0.74–1.57]	0.7 [0.27–1.85]

Statistically significant SSRs are in bold.

## Data Availability

No new data were created or analyzed in this study. Data sharing is not applicable to this article.

## Supplementary Materials

The following supporting information can be downloaded at: https://www.mdpi.com/article/10.3390/life13010120/s1, Table S1: Incidence rate ratios and their adjusted 95% CI for VTPs between age class, amenorrhea class, previous abortions class and phases; Table S2: Incidence rate ratios and their adjusted 95% CI for SAs between age class, amenorrhea class, previous abortions class and phases.
