# Supplementary material for: A Retrospective Observational Study to Assess the Effect of the COVID-19 Pandemic on Spontaneous and Voluntary Abortivity in the Apulia Region of Italy
Source: Life (Basel). 2022 Dec 31;13(1):120. doi: 10.3390/life13010120 (PMC9866677; doi:10.3390/life13010120)
Supplement: Supplementary file 1 [file life-13-00120-s001.zip › life-2121513-supplementary.pdf]

Table S1. Incidence Rate Ratios and their adjusted 95% CI for VTPs between age class, amenorrhea class, previous abortions class and phases.

**Parameter**	**IRR [CI95%]**
Age class (years)	
20-24 vs 25-29	0.84 [0.76-0.92]
20-24 vs 30-34	0.78 [0.72-0.86]
20-24 vs 35-39	0.79 [0.72-0.86]
20-24 vs 40-44	1.45 [1.31-1.62]
20-24 vs 45-49	11.4 [8.86-14.7]
20-24 vs ≤ 19	1.98 [1.76-2.23]
25-29 vs 30-34	0.94 [0.86-1.02]
25-29 vs 35-39	0.94 [0.86-1.02]
25-29 vs 40-44	1.74 [1.57-1.93]
25-29 vs 45-49	13.7 [10.6-17.6]
25-29 vs ≤ 19	2.37 [2.11-2.67]
30-34 vs 35-39	1.00 [0.92-1.09]
30-34 vs 40-44	1.86 [1.68-2.05]
30-34 vs 45-49	14.6 [11.3-18.7]
30-34 vs ≤ 19	2.53 [2.26-2.84]
35-39 vs 40-44	1.85 [1.67-2.05]
35-39 vs 45-49	14.5 [11.3-18.7]
35-39 vs ≤ 19	2.53 [2.25-2.83]
40-44 vs 45-49	7.84 [6.07-10.1]
40-44 vs ≤ 19	1.36 [1.20-1.55]
45-49 vs ≤ 19	0.17 [0.13-0.23]
Amenorrhea class (weeks)	
9-10 vs 11-12	4.82 [4.33-5.37]
9-10 vs ≥ 13	4.50 [4.04-5.00]
9-10 vs ≤ 8	0.51 [0.48-0.54]
11-12 vs ≥ 13	0.93 [0.81-1.07]
11-12 vs ≤ 8	0.11 [0.10-0.12]
≥ 13 vs ≤ 8	0.11 [0.10-0.13]
Previous abortions class (number)	
1 vs 2	2.75 [2.45-3.09]
1 vs 3	7.05 [5.89-8.45]
1 vs ≥ 4	10.6 [8.39-13.4]
1 vs 0	0.27 [0.25-0.29]
2 vs 3	2.56 [2.10-3.12]
2 vs ≥ 4	3.85 [3.00-4.93]
2 vs 0	0.10 [0.09-0.11]
3 vs ≥ 4	1.50 [1.13-1.99]
3 vs 0	0.04 [0.03-0.05]
≥ 4 vs 0	0.03 [0.02-0.03]
Phase	
Ph 1 vs Ph 2	0.63 [0.57-0.69]
Ph 1 vs Ph 3	0.72 [0.65-0.80]
Ph 1 vs Ph 4	0.15 [0.14-0.16]
Ph 2 vs Ph 3	1.14 [1.04-1.25]
Ph 2 vs Ph 4	0.24 [0.22-0.26]
Ph 3 vs Ph 4	0.21 [0.19-0.23]

Table S2. Incidence Rate Ratios and their adjusted 95% CI for SAs between age class, amenorrhea class, previous abortions class and phases.

Parameter	IRR [CI95%]
Age class (years)	
20-24 vs 25-29	0.48 [0.41-0.56]
20-24 vs 30-34	0.26 [0.23-0.30]
20-24 vs 35-39	0.23 [0.20-0.27]
20-24 vs 40-44	0.35 [0.30-0.41]
20-24 vs 45-49	2.34 [1.84-2.98]
20-24 vs ≤ 19	3.43 [2.57-4.59]
25-29 vs 30-34	0.55 [0.49-0.61]
25-29 vs 35-39	0.49 [0.44-0.54]
25-29 vs 40-44	0.73 [0.65-0.82]
25-29 vs 45-49	4.91 [3.93-6.13]
25-29 vs ≤ 19	7.20 [5.48-9.47]
30-34 vs 35-39	0.89 [0.81-0.97]
30-34 vs 40-44	1.34 [1.21-1.48]
30-34 vs 45-49	8.98 [7.25-11.1]
30-34 vs ≤ 19	13.2 [10.1-17.2]
35-39 vs 40-44	1.51 [1.37-1.66]
35-39 vs 45-49	10.1 [8.18-12.5]
35-39 vs ≤ 19	14.8 [11.4-19.4]
40-44 vs 45-49	6.72 [5.41-8.35]
40-44 vs ≤ 19	9.85 [7.52-12.9]
45-49 vs ≤ 19	1.47 [1.06-2.04]
Amenorrhea class (weeks)	
9-10 vs 11-12	2.68 [2.44-2.94]
9-10 vs ≥ 13	3.77 [3.39-4.19]
9-10 vs ≤ 8	0.91 [0.85-0.97]
11-12 vs ≥ 13	1.41 [1.24-1.59]
11-12 vs ≤ 8	0.34 [0.31-0.37]
≥ 13 vs ≤ 8	0.24 [0.22-0.27]
Previous abortions class (number)	
1 vs 2	3.11 [2.68-3.60]
1 vs 3	8.50 [6.71-10.8]
1 vs ≥ 4	12.8 [9.36-17.6]
1 vs 0	0.26 [0.24-0.29]
2 vs 3	2.74 [2.11-3.55]
2 vs ≥ 4	4.13 [2.96-5.76]
2 vs 0	0.09 [0.07-0.10]
3 vs ≥ 4	1.51 [1.03-2.21]
3 vs 0	0.03 [0.02-0.04]
≥ 4 vs 0	0.02 [0.02-0.03]
Phase	
Ph 1 vs Ph 2	0.49 [0.43-0.56]
Ph 1 vs Ph 3	0.55 [0.49-0.63]
Ph 1 vs Ph 4	0.12 [0.11-0.14]
Ph 2 vs Ph 3	1.13 [1.01-1.25]
Ph 2 vs Ph 4	0.25 [0.23-0.27]
Ph 3 vs Ph 4	0.22 [0.20-0.24]
